# Free and Bound Phenolics of Buckwheat Varieties: HPLC Characterization, Antioxidant Activity, and Inhibitory Potency towards *α*-Glucosidase with Molecular Docking Analysis

**DOI:** 10.3390/antiox8120606

**Published:** 2019-11-29

**Authors:** Huilan Zhu, Sixin Liu, Linling Yao, Lu Wang, Congfa Li

**Affiliations:** College of Food Science and Engineering, Hainan University, Haikou 570228, China; 13016276276@139.com (H.Z.); sixinliu@126.com (S.L.); Yaoll955@163.com (L.Y.)

**Keywords:** buckwheat varieties, free phenolic, bound phenolic, antioxidant activity, α-glucosidase inhibitory activity, molecular docking

## Abstract

Free and bound phenolic fractions from six buckwheat varieties were investigated for their compositions, antioxidant activities, and inhibitory effects on α-glucosidase. The results showed that different buckwheat varieties have significant differences in phenolic/flavonoid contents, and these contents were found in higher quantities in free form than in bound form. HPLC results revealed that rutin, quercetin, and kaempferol-3-O-rutinoside were the most abundant components in free and bound forms, whereas dihydromyricetin was found only in the bound form. Free phenolics showed higher antioxidant activities of DPPH, ABTS^+^, OH•, and FRAP than those of bound phenolics. Strong inhibitory effects against α-glucosidase by the free/bound phenolic fractions were found in all buckwheat varieties, and free phenolics showed stronger *α*-glucosidase inhibition than that of the corresponding bound phenolics. More importantly, the main phenolic compounds observed in the buckwheat varieties were subjected to molecular docking analysis to provide insight into their interactions with α-glucosidase. The contributions by individual phenolics to the observed variation was analysed by Pearson correlation coefficient analysis and principal component analysis. The present study provides a comprehensive comparison for the phenolic fractions of buckwheat varieties and identify the main contributors to antioxidant and α-glucosidase inhibitory activity.

## 1. Introduction

Nowadays, the incidence of conditions related to metabolic syndrome is rapidly rising worldwide, including oxidative damage, high blood sugar, hypertension, hyperlipidaemic diseases, and so on [1,2]. Nutritionists have confirmed that oxidative stress and high-calorie diets were closely related to the occurrence of chronic metabolic syndrome [3]. Chemical drugs are somewhat effective in relieving these chronic diseases, but they can also cause drug dependence and serious side effects [4]. Phytochemicals, including phenolics/flavonoids, which are found in cereal foods or natural products, have been demonstrated to have important physiological functions, such as weight loss and antioxidant, anti-diabetic, anti-inflammatory, anti-glycosylation, and anti-proliferative effects [5,6,7,8].

Buckwheat (*Fagopyrum esculentum* Moench), as an important functional cereal food of the *Polygonum* family, is widely distributed in Asia, Europe, Africa, North America, and Oceania [9]. Generally, buckwheat includes two species: common buckwheat (*Fagopyrum esculentum* Moench) and tartary buckwheat (*Fagopyrum tataricum* Gaertn) [10]. Buckwheat has received much attention not only because of its delicious flavor and nutritional quality in terms of macro-nutrients, but also as a cereal raw material rich in flavonoid compounds, which may reduce chronic conditions including oxidative damage, diabetes, and hypertensive diseases [11,12,13]. Researchers have reported that flavonoid contents in buckwheat were 23–45 and 25–50 times greater than those in wheat and corn, respectively [14,15]. Moreover, the phytochemical composition of cereal crops mainly depends quantitatively and qualitatively on their genotypes and environmental factors that affect growth [16,17]. Although many studies have measured the total phenolic contents and antioxidant capacity in some buckwheat varieties [12,18], information remains limited regarding the characterization and contents of free phenolic (FP) and bound phenolic (BP) fractions of different buckwheat varieties and their corresponding in vitro biological activities (especially anti-diabetic effects). Furthermore, the contributions of the total phenolic contents (TPC), total flavonoid contents (TFC), and the content of individual phenolic on their bio-activities has not been clearly investigated.

The aim of the present work was to systematically investigate the HPLC characterizations, in vitro antioxidant activities, and inhibitory effects against α-glucosidase of FP and BP fractions from six buckwheat varieties. More importantly, the potential inhibitory mechanism against α-glucosidase by the main phenolic compounds in six buckwheat samples was clarified by molecular docking analysis. In addition, the contributions of the individual phenolics to the observed variation were analyzed by Pearson correlation coefficient analysis and principal component analysis. This work may provide a comprehensive comparison for the phenolic fractions of buckwheat varieties and identify the main contributors to antioxidant and *α*-glucosidase inhibitory activity.

## 2. Materials and Methods

### 2.1. Materials and Reagents

Six fresh buckwheat samples, named S1–S6, were collected from China in August 2018. All samples have not been broken, damaged, or spoiled. The buckwheat varieties were identified by professional researches. All phenolic standards (HPLC-grade, >99.7%) were obtained from Nanjing Herbal Origin Biotechnology Co., Ltd. (Nanjing, Jiangsu, China). ABTS (2-azino-bis [3-ethylbenzothiazoline-6-sulfonic acid] diammonium salt), DPPH (1,1-diphenyl-2-picrylhydrazyl), TPTZ (2,4,6-tris(2-pyridyl]-s-triazine), and Trolox were purchased from Sigma-Aldrich Chemical Co., Ltd. (St. Louis, MO, USA). Formic acid and acetonitrile were purchased from Fisher Scientific (Waltham, MA, USA); all were HPLC-grade. Other analytical-grade reagents used in the study were purchased from Guangzhou Damou Chemical Reagent Co., Ltd. (Guangzhou, China).

### 2.2. Extraction of FP and BP Fractions

Different buckwheat samples were first freeze-dried with vacuum freeze dryer FD-2B-80 (Shanghai Gipp Electronic Technology Co., Ltd., Shanghai, China). Then, the dried samples were ground to a fine powder by a micronizer and sifted through a 20-mesh sieve. FP and BP fractions were extracted following the reported method [19]. In brief, 2 g of the above powder samples were blended with 8 mL of 80% ethanol in a 15 mL tube. The mixture was kept in a water bath at 50 °C for 30 min and then centrifuged at 4000× *g* for 10 min at 4 °C. The procedure was repeated twice, and then the filtrate was combined. After FP extraction, the residues were used to extract BP. One gram of the above dried residues was hydrolyzed by adding 40 mL of 2 M NaOH at 30 °C for 4 h under a nitrogen atmosphere. Then, the resultant hydrolysate was acidified to pH 2 with 6 M hydrochloric acid. The mixture was first degreased three times with 100 mL hexane. The supernatants were combined, extracted three times with the solvents (diethyl ether:ethyl acetate = 1:1, *v/v*), and then evaporated under reduced pressure at 30 °C. After removing the diethyl ether and ethyl acetate, the samples were dissolved in 5 mL of 50% ethanol (*v/v*) to obtain BP fractions, which were stored at −20 °C before analysis.

### 2.3. Determination of Phenolic and Flavonoid Contents

The Folin–Ciocalteau method was applied to determine phenolic content of different fractions [20]. Gallic acid was employed as standard. The average value of triplicate data was expressed as mg of gallic acid equivalents per gram sample in dry weight (mg GAE/g DW).

Flavonoid content in both free and bound phenolic fractions were quantified according to the aluminum chloride colorimetric method [21]. Rutin was employed as the standard. The average value of triplicate data was expressed as mg rutin equivalents per gram sample in dry weight (mg RE/g DW).

### 2.4. Quantitative Analysis by HPLC

A Hitachi 1200 HPLC system (SHIMADZU, Kyoto, Japan) was used to separate and quantify the phenolic compounds in free and bound phenolic fractions from BW samples. The HPLC system was equipped with a diode array detector (SPD-10A, SHIMADZU). Chromatographic separation was achieved in a Zorbax Eclipse Plus C18 column (250 mm × 4.6 mm, 5 μm, Agilent, Santa Clara, CA, USA). The mobile phase used was 0.1% formic acid-H2O (phase A) and 0.1% formic acid-acetonitrile (phase B) with the gradient program as follows: 15% B at 0–5 min, 15–20% B at 5–10 min, 20–50% B at 10–40 min, 50–80% B at 40–55 min, and 15% B at 55–60 min. The flow-rate was kept at 0.8 mL/min at all times. The other chromatographic conditions used were as follows: the column was operated at 30 °C, the scanning detection wavelength ranged from 200 to 600 nm, and the injection volume was 10.0 μL. All samples were analyzed in triplicate, and the amount of phenolic compound was expressed as micrograms per gram sample in dried weight (μg/g DW).

### 2.5. Antioxidant Activity Assays

#### 2.5.1. DPPH Radical Scavenging Activity Assay

The scavenging activity of DPPH free radicals was performed according to the reported method [22]. Briefly, 50 µL of the diluted sample extracts was mixed with 400 µL of 100 µM DPPH-methanol solution for 30 min at 25 °C. The absorbance was measured at 517 nm. Trolox solution (0–40 μg/mL) was used as the positive control. The results were expressed as µmol Trolox equivalents (TE)/g sample in DW (µmol TE/g DW).

#### 2.5.2. ABTS^+^ Radical Scavenging Activity Assay

The scavenging activity of ABTS^+^ radical was measured according to the previously described method [23]. Trolox solution (0–40 μg/mL) was used as the positive control. The ABTS^+^ value was expressed as µmol Trolox equivalents (TE)/g sample in DW (µmol TE/g DW).

#### 2.5.3. Hydroxyl (OH•) Radical Scavenging Activity Assay

The scavenging activity of OH• radical was measured using the reported method [24]. Briefly, 100 µL of the extract dilutions was mixed with 100 µL of 6 mM FeSO_4_ solution and 100 µL of 2.4 mM H_2_O_2_ solution. After incubating for 10 min at 25 °C, 100 µL of 6 mM salicylic acid was added to the reaction solution. The mixture was further incubated for 30 min at 25 °C; then, the absorbance was read at 510 nm. Trolox solution (0–40 μg/mL) was used as the positive control. The OH• value was expressed as µmol TE/g DW.

#### 2.5.4. Ferric Reducing Antioxidant Power (FRAP) Assay

FRAP assay was performed on the basis of the described method [25]. Ferrous sulfate solution (0, 50, 200, 400, 600, 800, and 1000 M) was used to establish the standard curve. The FRAP values were expressed in µM ferrous sulfate equivalents per gram sample in dried weight (µM Fe(II)SE/g DW).

### 2.6. Determination of α-Glucosidase Inhibitory Activity

The α-glucosidase inhibitory activity of FP and BP fractions in different samples was measured according to the previously described method [26]. Firstly, 100 µL of 1 U/mL α-glucosidase in phosphate buffer solution (PBS, 0.1 M, pH 6.8) was mixed with 50 µL of the test extracts dilutions in a 2 mL tube. After incubation at 37 °C for 10 min, the reaction was begun by adding 100 µL of 5 mM p-NPG solution, and incubated for 20 min at 37 °C. The absorbance of the reaction solution was read at 405 nm in 15 min. The inhibiting activity of α-glucosidase (%) was calculated on the basic of the following equation:

$$\alpha \text{-Glucosidase inhibition activity }(\%)\;=\;[\frac{\Delta A_{\mathrm{cont}}-{\Delta A}_{s}}{\Delta A_{\mathrm{cont}}}]\;\times \;100\%$$

where ΔA_cont_ = A_buffer+enzyme_ − A_buffer_, and ΔA_s_ = A_extract+enzyme_ − A_extract._

### 2.7. Molecular Docking Analysis

The ChemBio3D Ultra (CambridgeSoft Corporation, Massachusetts, United States) was used to draw the 2D structures of the main phenolic compounds in BW samples. It is worth noting that little information was available regarding the structure of *α*-glucosidase from *Saccharomyces cerevisiae*. Hence, its homologous structure (isomaltase, PDB ID: 3A4A) obtained from RCSB PDB was usually applied to conduct the docking test [27,28]. The Surflex-Dock Geom (SFXC) mode was used to perform docking analysis using SYBYL-X 2.0 software (Tripos, Inc., St. Louis, MO, USA). A docking score file was generated, and a C-score ≥ 4 was considered as a credible result. Several parameters including four score functions (T-score, PMF-score, D-score, and CHEM-score), hydrogen bond distances, and amino acid binding sites were used to explain the active inhibitory mechanisms of the main phenolics against *α*-glucosidase [29].

### 2.8. Statistical Analysis

All assays were conducted in triplicate. All values were expressed as the average value ± standard deviation (SD). SPSS Statistics version 17.0 (IBM SPSS, Chicago, IL, USA) was used to perform the statistical analyses. IC_50_ value was measured by Probit analysis on SPSS Statistics version 17.0. Significant differences (*p* < 0.05) were considered statistically significant. Correlation analysis between the analytes and the investigated bio-activities were evaluated using Pearson correlation.

## 3. Results and Discussion

### 3.1. Total Phenolic Contents (TPC) and Total Flavonoid Contents (TFC)

As shown in Table 1, significant differences were observed with respect to TPC and TFC in different buckwheat samples. The contents of free phenolic (FP) and free flavonoid (FF) in six buckwheat samples ranged between 5.18–13.74 mg GAE/g DW and 7.37–26.60 mg RE/g DW, respectively, while bound phenolic (BP) and bound flavonoid (BF) contents ranged between 0.63 and 0.96 mg GAE/g DW and 0.72 and 1.38 mg RE/g DW, respectively. It was found that FP and FF were the main contributors to TPC/TFC, accounting for over 90% of contents. Moreover, the FP/FF contents and TPC/TFC of the buckwheat sample from Shanxi were significantly higher (*p* < 0.05) than those of the samples from other genotypes and regions in China (Table 1).

Qin et al. (2010) reported that the FP contents (25.3 mg GAE/g DW) were higher than the BP contents (1.8 mg GAE/g DW) in tartary buckwheat bran [15]. Liu et al. (2019) confirmed that the highest phenolic content of 15 buckwheat varieties from China was only 7.32 mg GAE/g DW, which was lower than that of samples from Shanxi (13.74 mg GAE/g DW) [30]. In this work, we found that the average TPC and TFC of tartary buckwheat samples (TPC: 9.97 mg GAE/g DW; TFC: 19.26 mg RE/g DW) were significantly higher than those of the common buckwheat samples (TPC: 6.47 mg GAE/g DW; TFC: 10.87 mg RE/g DW) (*p* < 0.001). Owing to the genotypes and growth-influencing environmental factors of buckwheat varieties, significant differences were seen in TPC/TFC. Many studies have confirmed that phytochemical compositions of cereal crops mainly depend qualitatively and quantitatively on its genotypes and environmental factors that influence growth [30,31].

### 3.2. Quantitative HPLC Analysis of Phenolic Compositions

**Figure 1:**
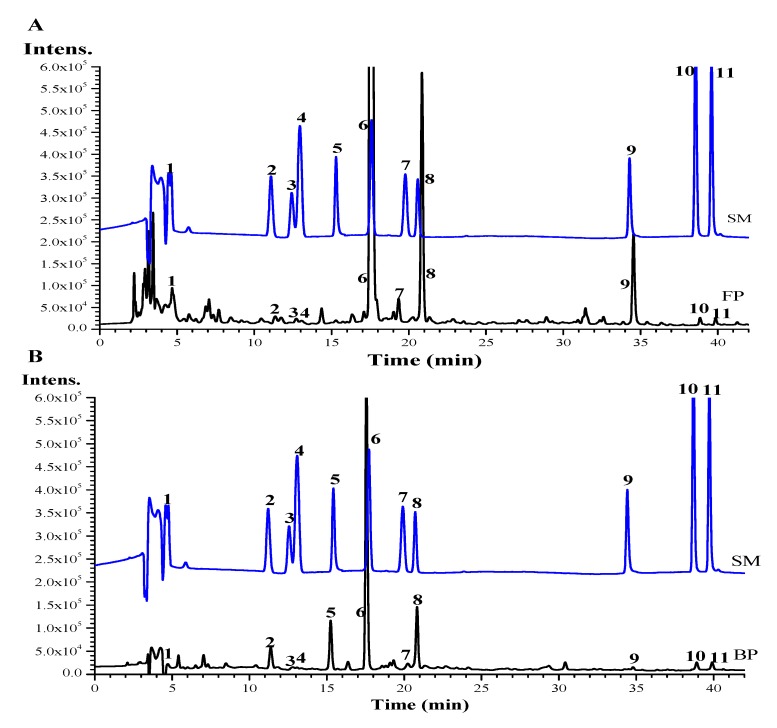
HPLC chromatograms (280 nm) of free (**A**) and bound phenolic fractions (**B**) of buckwheat. Peaks identification and their MS data are shown in Table S1. 1, Gallic acid; 2, 4-hydroxybenzoic acid; 3, 5-caffeoylquinic acid; 4, syringic acid; 5, dihydromyricetin; 6, rutin; 7, ferulic acid; 8, kaempferol-3-O-rutinoside; 9, quercetin; 10, apigenin; 11, kaempferol.

Phenolic compositions were preliminary identified by comparing retention time (RT), UV spectra, and the MS spectral data of standards (Table S1). The phenolic compositions of FP and BP fractions from six buckwheat samples were quantified by HPLC (Figure 1). As shown in Figure 1 and Table 2, it can be seen that phenolic compounds in buckwheat samples were divided into two categories: flavonoids and phenolic acid groups. The six flavonoid compounds included rutin, dihydromyricetin, kaempferol-3-*O*-rutinoside, quercetin, apigenin, and kaempferol. Five of these compounds existed in free and bound forms, except dihydromyricetin, which was only detected in BP fractions. Five phenolic acids including gallic acid, 4-hydroxybenzoic acid, 5-caffeoylquinic acid, syringic acid, and ferulic acid were detected in the free and bound fractions. Among them, rutin, kaempferol-3-O-rutinoside, and quercetin were the most predominant compounds that existed in both forms.

Regarding FP, the Shanxi sample included significantly higher (*p* < 0.05) rutin (6288.26 µg/g), kaempferol-3-O-rutinoside (3618.65 µg/g), and quercetin (1379.54 µg/g) than the other samples, which resulted in higher FPC and TPC (*p* < 0.05).

Significantly higher quantities of rutin, kaempferol-3-O-rutinoside, ferulic acid, quercetin, apigenin, and kaempferol were found in free form than those in bound form, which was consistent with the report described by Li et al. (2016) [10]. However, some phenolic acid compounds including gallic acid, 4-hydroxybenzoic acid, 5-caffeoylquinic acid, and syringic acid were significantly more common in bound form. Samples from Shangdong and Heilongjiang contained high bound gallic acid and syringic acid. Many studies have confirmed that rutin, quercetin, and isoquercitin were the most predominant compounds in buckwheat [32]. In the present work, in addition to quercetin and rutin, high kaempferol-3-O-rutinoside contents were also found in all buckwheat samples, which may be due to differences in genotypes and growth-influencing environmental factors [31].

### 3.3. Antioxidant Activities

Albishi et al. (2013) suggested that at least two test methods should be used to evaluate the in vitro antioxidant activity of samples, owing to different mechanisms involved in determining antioxidant capacity [33]. In this study, four independent methods including FRAP and the radical scavenging activities of DPPH, ABTS^+^, and OH• were used to comprehensively evaluate the antioxidant capacity of the FP and BP fractions from different buckwheat varieties.

The results showed that the antioxidant capacities of FP fractions of six buckwheat varieties varied significantly (*p* < 0.05). Meanwhile, DPPH levels of FP and BP fractions in different buckwheat samples ranged between 17.55–114.02 µmol TE/g DW and 4.30–7.68 µmol TE/g DW, respectively. For ABTS^+^ assays, FP and BP fractions yielded ABTS^+^ values of 69.19–175.66 µmol TE/g DW and 7.12–10.62 µmol TE/g DW, respectively. However, OH• levels of FP fractions in different buckwheat samples ranged between 32.92–82.64 µmol TE/g DW. The OH• values of BP fractions showed no significant differences (*p* > 0.05) among the different buckwheat samples. FRAP values of FP fractions in different buckwheat samples ranged between 29.58 and 84.72 mM FeS(II)E/g DW (Table 3). The highest antioxidant capacity of FP fractions was detected in the buckwheat sample from Shanxi (DPPH: 114.02 µmol TE/g DW; ABTS^+^: 175.66 µmol TE/g DW; OH•: 82.64 µmol TE/g DW; FRAP: 84.72 mM FeS(II)E/g DW), which was due to its high TPC/TFC and individual phenolic contents. Moreover, the antioxidant activities of FP fractions from different buckwheat samples were significantly higher than those of the BP fractions. More importantly, it was found that the average antioxidant activities (DPPH: 71.54 µmol TE/g DW; ABTS^+^: 138.48 µmol TE/g DW; OH•: 73.39 µmol TE/g DW; FRAP: 58.41 mM FeS(II)E/g DW) in tartary buckwheat samples were higher than those in common buckwheat samples (DPPH: 33.79 µmol TE/g DW; ABTS^+^: 89.05 µmol TE/g DW; OH•: 55.65 µmol TE/g DW; FRAP: 37.03 mM FeS(II)E/g DW). The results showed that FP contributes to the main antioxidant activities in buckwheat samples. Li et al. (2016) also confirmed that the FP fractions of buckwheat bran samples contributed to the main antioxidant activities [10]. In addition, higher phenolic contents resulted in stronger antioxidant activities. Xiang et al. (2019) reported that the phenolic contents of finger millets have a strong positive correlation with the oxygen radical absorbance capacity and ABTS^+^ radical scavenging activities (*r* = 0.948, *r* = 0.836, respectively; *p* < 0.01) [34].

### 3.4. Inhibitory Activity against α-Glucosidase

Some chemical drugs are widely applied to manage type II diabetes, but some side effects have been reported in their application [35]. Consequently, there is an urgent need to identify natural alternative products without side effects to manage type II diabetes. Many studies have confirmed that *α*-glucosidase inhibitors from cereal products, which had fewer side effects, played important roles in regulating blood glucose levels [36,37].

**Figure 2:**
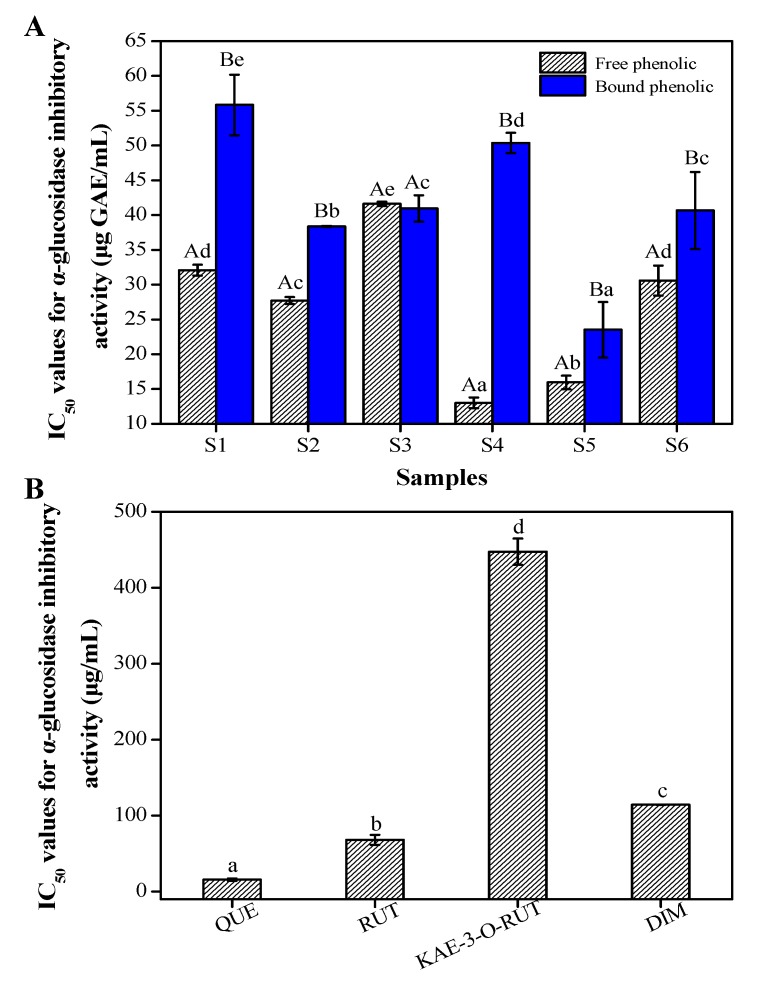
The α-glucosidase inhibitory activity (IC_50_) of free and bound phenolic fractions (**A**) in different buckwheat samples and their main phenolics molecules (**B**). QUE, quercetin; RUT, rutin; KAE-3-O-RUT, Kaempferol-3-O-rutinoside. DIM, dihydromyricetin. Different uppercase letters (**A**,**B**) mean statistically significant differences in free and bound phenolic fractions of different samples. Different lowercase letters (a–e) mean statistically significant differences following different samples/analytes at the same fraction.

Figure 2A demonstrates that FP and BP fractions in different buckwheat samples tended to be strong *α*-glucosidase inhibitors. Moreover, these fractions all showed inhibitory activity against *α*-glucosidase in a concentration-dependent manner. It is worth noting that higher IC_50_ values indicated lower *α*-glucosidase inhibition. Furthermore, the highest *α*-glucosidase inhibition in FP fractions was found in Shanxi (IC_50_ = 13.00 ± 0.75 μg GAE/mL) and Shangdong (IC_50_ = 15.94 ± 0.98 μg GAE/mL) samples. The BP fractions of the Shangdong sample also showed strong *α*-glucosidase inhibition (IC_50_ = 23.51 ± 4.01 μg GAE/mL). Figure 2B displays the IC_50_ values for *α*-glucosidase inhibition by the main phenolic compounds from the buckwheat samples. It can be seen that the IC_50_ of quercetin was 15.71 ± 1.43 μg/mL, which was higher than those of rutin (68.16 ± 3.61 μg/mL), kaempferol-3-O-rutinoside (447.50 ± 17.27 μg/mL), and dihydromyricetin (114.45 ± 0.31 μg/mL) (*p* < 0.01). Many studies have stated that the inhibitory activities against digestive enzymes by cereal foods are due to phenolic/flavonoid compounds [38,39]. Wang et al. (2018) confirmed that flavonoid compounds, especially quercetin, possessed strong capacities for *α*-glucosidase inhibition [26]; Qin et al. (2013) also reported such a strong capacity by rutin in tartary buckwheat [40]. For buckwheat samples, higher flavonoid contents (rutin and quercetin) resulted in a stronger *α*-glucosidase inhibitory capacity.

### 3.5. Molecular Docking Analysis

**Figure 3:**
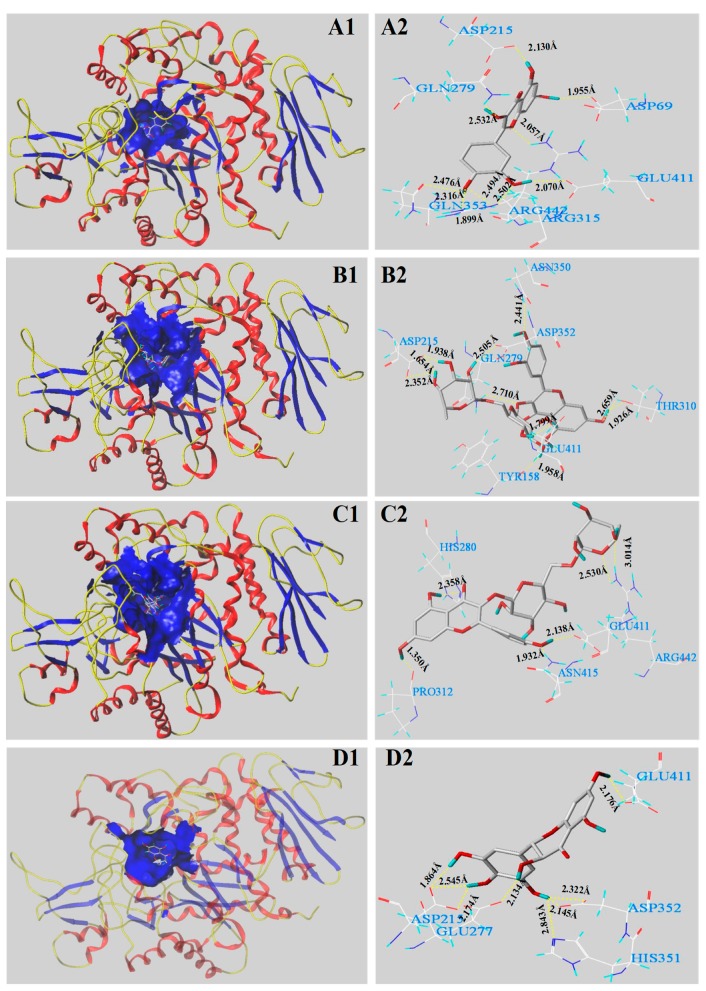
Molecular docking of four main phenolic compounds with the *α*-glucosidase. The 3D docking structures of four phenolic compounds were inserted into the hydrophobic cavity of the *α*-glucosidase (blue): quercetin (**A1**); rutin (**B1**); kaempferol-3-*O*-rutinoside (**C1**); dihydromyricetin (**D1**). The conformation of active molecules interactions with amino acid residues in the active site of *α*-glucosidase: quercetin (**A2**); rutin (**B2**); kaempferol-3-*O*-rutinoside (**C2**); dihydromyricetin (**D2**) with residues in the active sites of the *α*-glucosidase, respectively. The yellow dashed line represented hydrogen bonds.

In the present work, the inhibitory mechanisms of the main four phenolic constituents in BW samples including quercetin, rutin, kaempferol-3-O-rutinoside, and dihydromyricetin against *α*-glucosidase were further illuminated by molecular docking analysis. Figure 3 and Table 4 show the docking results regarding interactions between the main phenolic molecules and *α*-glucosidase binding. As shown in Table 4, all four main phenolic molecules had C-scores ≥ 4, indicating reliable docking values. The T-score function, an important docking parameter, is a weighted sum of non-linear functions involving van der Waals surface distances between the ligand atoms and exposed receptor enzymes [27,29]. Quercetin, with a docking T-score of 6.37, exhibited strong hydrogen bonding interactions with *α*-glucosidase and formed ten H-bonds with the seven catalytic residues of Asp 69, Asp 215, Arg 315, Arg 442, Gln 353, Glu 411, and Gln 279 of the *α*-glucosidase receptor (Figure 3A1,A2 and Table 4). The H-bond distances ranged from 1.899 to 2.532 Å. Rutin, with a docking T-score of 5.94, formed ten H-bonds within 4 Å (distances of 1.654–2.710 Å) with seven amino acid catalytic residues (Asp 215, Asp 352, Asn 350, Tyr 158, Tyr 310, Glu 411, and Gln 279) of *α*-glucosidase (Figure 3B1,B2 and Table 4). Six H-bonds with five amino acid residues (Pro 312, Asn 415, Arg 442, Glu 411, and His 280) were observed for kaempferol-3-O-rutinoside, with a docking score of 4.68 (Figure 3C1,C2 and Table 4). The average H-bond distance was 2.220 Å. The docking score of dihydromyricetin was 5.32, indicating eight H-bond interactions with five active site residues (Asp 215, Asp 352, Glu 277, Glu 411, and His 351). The distances ranged from 1.864 to 2.843 Å (Figure 3D1,D2 and Table 4).

The results clearly showed that the phenolic compound structures influenced the inhibitory effects on *α*-glucosidase. Among them, the numbers of H-bonds and active sites residues played important roles in exerting the catalytic functions of the complex of the *α*-glucosidase receptor and ligands. When the main four phenolics were docked with *α*-glucosidase, the numbers of formed active site residues were ordered as follows: quercetin (7) = rutin (7) > dihydromyricetin (5) = kaempferol-3-O-rutinoside (5); those of the formed H-bonds were as follows: quercetin (10) = rutin (10) > dihydromyricetin (8) > kaempferol-3-O-rutinoside (6). As a result, quercetin showed the strongest *α*-glucosidase inhibition (Figure 2B). Although quercetin and rutin docked with *α*-glucosidase exhibited equal numbers of H-bonds and active site residues, there were significant differences in the capacity for *α*-glucosidase inhibition. This may be because these different molecules had different residue interaction sites with *α*-glucosidase. Both quercetin and rutin interacted with the amino acid residues Glu 411 and Gln 279, indicating that these two residues may be the important catalytic sites of *α*-glucosidase. However, kaempferol-3-O-rutinoside formed an H-bond with Asp 415, indicating that it may bind to the active site of *α*-glucosidase to inhibit its catalytic activity. Consequently, kaempferol-3-O-rutinoside exhibited the weakest inhibitory effect against *α*-glucosidase. It was found that Glu 411 bound with each of the four phenolics, implying that it may exert important functions in the catalytic reaction of *α*-glucosidase. Many studies also verified that Asp 215 and Glu 411 were the important active sites involved in this catalytic reaction [28,34]. In addition, the formation of hydrogen bonds between the hydroxyl group at C-3 or C-4′ of the molecules (i.e., quercetin and rutin) and the active site residues may produce a higher inhibitory ability towards *α*-glucosidase compared to kaempferol-3-O-rutinoside, which was consistent with the results reported by Zeng et al. (2016) [41]. Rasouli et al. (2017) also reported that the hydrogen bonds and active site residues formed by the ligand molecules and receptor enzymes exerted important effects on *α*-glucosidase inhibitory activities [42].

### 3.6. Correlations between the Investigated Bio-Activities and Phenolic Compositions

To explore the effect of the phenolic compounds on the investigated bio-activities in different buckwheat varieties, correlations among the examined variables were elucidated by Pearson correlation coefficient analysis [43].

As shown in Table 5, correlation coefficients were determined for FP vs. DPPH (*r* = 0.990, *p* < 0.001), ABTS^+^ (*r* = 0.983, *p* < 0.01), OH• (*r* = 0.851, *p* < 0.05), FRAP (*r* = 0.998, *p* < 0.001), and *α*-glucosidase inhibitory activity (*r* = 0.671, *p* < 0.05). BP fractions of buckwheat were also significantly correlated to DPPH (*r* = 0.583, *p* < 0.05), ABTS^+^ (*r* = 0.932, *p* < 0.01), OH^−^ (*r* = 0.803, *p* < 0.05), FRAP (*r* = 0.947, *p* < 0.01), and *α*-glucosidase inhibitory activity (*r* = 0.604, *p* < 0.05). The antioxidant activities were also significantly positive correlated to FF and BF contents (*p* < 0.05). Meanwhile, the antioxidant activities including DPPH, ABTS^+^, OH•, and FRAP values were significantly positively correlated to gallic acid, rutin, dihydromyricetin, quercetin, and kaempferol-3-O-rutinoside contents (*p* < 0.05). A positive correlation was detected between FP contents and *α*-glucosidase inhibitory activity (*r* = 0.671, *r* = 0.723, *p* < 0.05). Inhibitory activity against *α*-glucosidase was also significantly correlated to dihydromyricetin, rutin, kaempferol-3-O-rutinoside, quercetin, and kaempferol contents (*r* = 0.765, 0.803, 0.551, 0.715, and 0.618, respectively; *p* < 0.05). Among them, three phenolic compounds including rutin, kaempferol-3-O-rutinoside, and quercetin contributed mainly to the investigated bio-activities of different buckwheat varieties, whereas rutin, kaempferol-3-O-rutinoside, and dihydromyricetin contributed to the bio-activities of BP fractions among the varieties. It is worth noting that the correlation analysis results will give more reliable results if a greater number of samples were obtained.

### 3.7. Principal Component Analysis (PCA)

PCA is widely used to reduce the dimensionality and increase the interpretability of large datasets. To systematically and fully investigate the contributions of the individual phenolics to the variables investigated, PCA was carried out using FP, BP, FF, BF, the individual phenolic contents, antioxidant activities (DPPH, ABTS^+^, OH•, and FRAP values), and *α*-glucosidase inhibitory activity (IC_50_) for different buckwheat samples.

**Figure 4:**
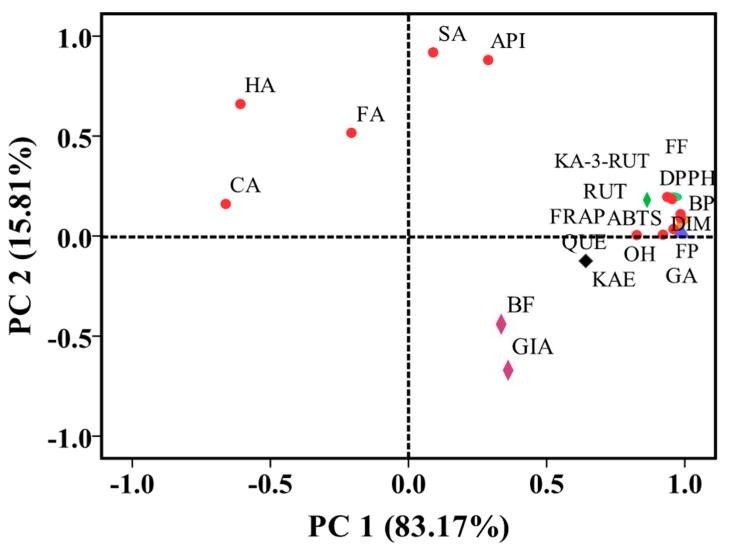
Loading plot of principal component analysis (PCA) from the variation observed of six buckwheat samples. FP, free phenolic; FF, free flavonoid; BP, bound phenolic; BF, bound flavonoid; GA, gallic acid; HA, 4-hydroxybenzoic acid; CA, 5-caffeoylquinic acid; SA, syringic acid; DIM, dihydromyricetin; RUT, rutin; FA, ferulic acid; KAE-3-RUT, kaempferol-3-O-rutinoside; QUE, quercetin; API, apigenin; KAE, kaempferol; DPPH, 1,1-diphenyl-2-picrylhydrazyl; ABTS^+^, 2, 2-azino-bis (3-ethylbenzothiazoline-6-sulfonic acid) diammonium salt; FRAP, ferric reducing/antioxidant power; OH•, hydroxyl radical.

PCA yielded two principal components (with an eigenvalue higher than 1) explaining 98.98% of the total variances in the data to simplify the analysis of the results. The loading plot illustrates the relationship between the investigated variables (Figure 4). The two principal components PC1 and PC2 accounted for 83.17% and 15.81% of the total variation, respectively. Among them, PC1 separated the samples based on FP, BP, FF, DPPH, ABTS^+^, OH•, FRAP values, rutin, kaempferol-3-O-rutinoside, dihydromyricetin, and quercetin, which are present in the upper right square. The variables were separated along PC2 by differences observed in 4-hydroxybenzoic acid, ferulic acid, and 5-caffeoylquinic acid, which are present in the upper left square. The results demonstrated that FP, BP, FF, rutin, kaempferol-3-O-rutinoside, dihydromyricetin, and quercetin were closely correlated with DPPH, ABTS^+^, OH•, and FRAP values, which was consistent with the results of Pearson correlation coefficient analysis. Therefore, the scatter plot produced by PCA may be used to reduce the dimensionality and interpret the differences among the variables in large datasets.

## 4. Conclusions

In this study, the characterizations and contents of FP and BP fractions in different buckwheat varieties and their corresponding in vitro biological activities (especially antioxidant and anti-diabetic) were first reported. The results showed that the TPC and TFC of tartary buckwheat were significant higher than those of common buckwheat. Moreover, for all tartary buckwheat varieties, phenolic/flavonoid contents in free form were found in greater quantities than those in bound form. HPLC results revealed that rutin, quercetin, and kaempferol-3-O-rutinoside were the most abundant components found in free and bound forms, whereas dihydromyricetin was only found in BP. FP showed higher antioxidant activities of DPPH, ABTS^+^, OH•, and FRAP than those of BP. Among them, FP in buckwheat samples from Shanxi exhibited the highest antioxidant activity and inhibitory activity towards *α*-glucosidase. In addition, the strong inhibitory effects against *α*-glucosidase by FP and BP fractions in buckwheat varieties were illuminated by molecular docking analysis. The contributions of the individual phenolics to the investigated bio-activities were analyzed by Pearson correlation coefficient analysis and PCA. The present study demonstrated that phenolic fractions (especially free forms) of different buckwheat samples had strong antioxidant activities and inhibitory effects on *α*-glucosidase and provided evidence for the qualitative evaluation of buckwheat varieties.

## Figures and Tables

**Table 1:** Specific information, free and bound phenolic/flavonoid contents of the six buckwheat samples from China.

Abbre.	Collect Location	Type	Color	Phenolic Contents (mg GAE/g DW)			Flavonoid Contents (mg RE/g DW)		
				FP	BP	TP	FF	BF	TF
S1	Tianjin, China	Tartary buckwheat	Black	7.69 ± 0.35c	0.73 ± 0.04b	8.42 ± 0.39d	14.48 ± 0.26d	1.07 ± 0.04b	15.55 ± 0.30d
S2	Sichuan, China	Common buckwheat	Green black	5.33 ± 0.27a	0.96 ± 0.02c	6.29 ± 0.28b	7.37 ± 0.21a	1.17 ± 0.03b	8.54 ± 0.23a
S3	Yunnan, China	Common buckwheat	Black	6.57 ± 0.25b	0.74 ± 0.02b	7.31 ± 0.27c	11.88 ± 0.53c	1.28 ± 0.09c	13.16 ± 0.57c
S4	Shanxi, China	Tartary buckwheat	Light yellow	13.74 ± 0.24d	0.66 ± 0.03a	14.40 ± 0.25e	26.60 ± 0.86e	0.72 ± 0.01a	27.32 ± 0.79e
S5	Shangdong, China	Common buckwheat	Dark yellow	5.18 ± 0.25a	0.63 ± 0.03a	5.81 ± 0.27a	10.07 ± 0.12b	0.85 ± 0.03a	10.92 ± 0.12b
S6	Heilongjiang, China	Tartary buckwheat	Dark green	6.19 ± 0.01b	0.90 ± 0.03c	7.09 ± 0.05c	13.53 ± 0.85d	1.38 ± 0.04c	14.91 ± 0.87c

Different lowercase letters (a–e) mean statistically significant differences following different samples at the same fractions (*p* < 0.05). FP, free phenolic; BP, bound phenolic; TP, total phenolic; FF, free flavonoid; BF, bound flavonoid; TF, total flavonoid.

**Table 2:** Individual phenolic compounds contents in free and bound fractions of six buckwheat samples from China.

Analytes	Status	Contents (μg/g DW)					
		S1	S2	S3	S4	S5	S6
Gallic acid (GA)	FP	22.96 ± 5.4c	13.30 ± 2.60b	9.55 ± 1.56a	75.01 ± 1.53d	ND	ND
	BP	67.06 ± 0.82a	64.76 ± 0.75a	71.18 ± 3.12b	60.66 ± 1.07a	68.73 ± 0.38b	59.79 ± 1.00a
	TP	90.02 ± 4.99d	78.05 ± 3.32c	80.74 ± 4.90c	135.67 ± 1.31e	68.73 ± 0.38b	59.79 ± 1.00a
4-Hydroxybenzoic acid (4-HA)	FP	99.39 ± 0.71a	97.63 ± 1.07a	96.80 ± 0.40a	93.26 ± 0.55a	122.57 ± 1.23c	102.64 ± 8.18b
	BP	175.29 ± 6.65d	165.75 ± 3.58c	78.85 ± 5.33b	164.31 ± 8.44c	193.72 ± 10.89e	61.57 ± 7.28a
	TP	274.68 ± 6.53d	263.38 ± 2.55c	175.65 ± 4.95b	257.58 ± 8.04c	296.58 ± 12.12e	164.21 ± 6.72a
5-Caffeoylquinic acid (5-CA)	FP	4.20 ± 0.06b	6.73 ± 6.03c	17.68 ± 2.06d	ND	19.55 ± 0.71d	2.20 ± 0.09a
	BP	33.82 ± 2.43b	2.61 ± 0.99a	46.96 ± 0.01c	ND	ND	ND
	TP	38.02 ± 2.49d	9.65 ± 5.43b	64.64 ± 2.06e	ND	19.55 ± 0.71c	2.20 ± 0.09a
Syringic acid (SA)	FP	ND	19.39 ± 0.44a	ND	31.99 ± 3.35b	36.75 ± 0.51b	34.50 ± 7.91b
	BP	66.97 ± 2.43d	27.46 ± 1.97c	6.69 ± 0.47b	25.74 ± 2.76c	4.28 ± 0.16a	6.93 ± 1.21b
	TP	66.97 ± 2.43d	46.85 ± 1.96b	6.69 ± 0.47a	57.73 ± 4.90c	41.03 ± 0.64b	41.44 ± 6.90b
Dihydromyricetin (DIM)	FP	ND	ND	ND	ND	ND	ND
	BP	123.13 ± 12.37c	299.93 ± 1.43e	83.25 ± 0.57b	148.95 ± 0.42d	57.94 ± 0.50a	57.85 ± 0.14a
	TP	123.13 ± 12.37c	299.93 ± 1.43e	83.25 ± 0.57b	148.95 ± 0.42d	57.94 ± 0.50a	57.85 ± 0.14a
Rutin (RUT)	FP	3813.38 ± 110.33a	1294.63 ± 38.38b	3273.15 ± 86.34d	6288.26 ± 144.01f	2646.60 ± 142.33c	3409.12 ± 66.11e
	BP	244.75 ± 7.45b	236.66 ± 11.82b	324.15 ± 21.98c	85.02 ± 4.76a	222.05 ± 11.55c	416.83 ± 10.87d
	TP	4058.13 ± 107.06e	1531.29 ± 32.71a	3597.30 ± 64.89c	6373.28 ± 148.57f	2868.66 ± 140.69b	3825.95 ± 76.95d
Ferulic acid (FA)	FP	ND	9.26 ± 0.18a	12.77 ± 0.31b	9.69 ± 0.21a	10.95 ± 0.37a	12.39 ± 0.17b
	BP	3.88 ± 0.10a	3.41 ± 0.13a	3.71 ± 0.19a	6.32 ± 0.83b	3.15 ± 0.11a	3.08 ± 0.09a
	TP	3.88 ± 0.10a	12.67 ± 0.29b	16.49 ± 0.37c	16.01 ± 0.68c	14.10 ± 0.41b	15.46 ± 0.10c
Kaempferol-3-O-rutinoside (KAE-3-RUT)	FP	1639.34 ± 56.63d	511.06 ± 15.67a	1100.85 ± 31.49c	3618.65 ± 111.97e	979.31 ± 50.05b	1077.41 ± 21.29c
	BP	212.33 ± 6.29c	215.77 ± 13.92c	229.35 ± 20.47d	43.34 ± 1.34a	161.17 ± 13.32b	230.85 ± 16.41d
	TP	1851.67 ± 54.34d	726.83 ± 14.66a	1330.20 ± 12.43b	3661.99 ± 111.75d	1140.48 ± 49.60c	1146.31 ± 37.59c
Quercetin (QUE)	FP	262.14 ± 3.35b	872.93 ± 21.23d	328.32 ± 8.74c	1379.54 ± 33.82e	144.05 ± 1.68a	141.03 ± 2.03a
	BP	49.51 ± 0.38a	52.56 ± 2.91b	47.99 ± 0.16a	58.35 ± 0.17c	48.38 ± 0.03a	47.96 ± 0.11a
	TP	311.65 ± 3.69b	925.49 ± 21.13d	376.30 ± 8.61c	1437.89 ± 33.96e	192.43 ± 1.71a	188.99 ± 2.10a
Apigenin (API)	FP	110.25 ± 1.31b	108.97 ± 0.03a	108.86 ± 0.13a	114.07 ± 0.58b	113.94 ± 2.47b	113.70 ± 2.87b
	BP	70.58 ± 2.28a	77.41 ± 6.94b	79.33 ± 2.38b	82.69 ± 0.53b	82.53 ± 0.88b	79.25 ± 1.09b
	TP	180.83 ± 3.57a	186.38 ± 6.92b	188.19 ± 2.33b	196.76 ± 0.62c	196.48 ± 2.55c	192.95 ± 3.66b
Kaempferol (KAE)	FP	106.24 ± 0.21a	139.15 ± 1.38b	108.39 ± 0.39a	140.10 ± 1.12b	101.03 ± 0.05a	100.90 ± 0.08a
	BP	64.98 ± 0.31a	64.20 ± 0.17a	62.36 ± 0.07a	62.90 ± 0.02a	62.37 ± 0.02a	63.75 ± 0.17a
	TP	171.22 ± 0.52b	203.35 ± 1.22c	170.75 ± 0.42b	203.00 ± 1.10c	163.40 ± 0.07a	164.65 ± 0.09a

Different lowercase letters (a–e) mean statistically significant differences following different samples at the same status (*p* < 0.05). FP, free phenolic; BP, bound phenolic; TP, total phenolic. N.D. not detected.

**Table 3:** The antioxidant activities of free and bound phenolic fractions of different buckwheat samples.

Antioxidant Activities	Status	S1	S2	S3	S4	S5	S6
DPPH (μmol TE/g DW)	FP	44.97 ± 1.86d	17.55 ± 3.10a	37.35 ± 3.46c	114.02 ± 0.36e	26.69 ± 2.24b	39.38 ± 1.29 c
	BP	4.30 ± 0.51a	7.68 ± 0.20d	5.65 ± 0.43b	5.54 ± 1.16b	6.46 ± 1.84c	6.40 ± 0.35c
	TP	49.27 ± 2.37d	25.23 ± 3.30a	43.00 ± 3.89c	119.56 ± 1.52e	33.15 ± 4.08b	45.78 ± 1.64c
ABTS^+^ (μmol TE/g DW)	FP	119.12 ± 1.32d	69.19 ± 0.30a	93.50 ± 1.09c	175.66 ± 1.57e	75.80 ± 2.28b	92.12 ± 1.31c
	BP	9.01 ± 0.27a	11.54 ± 0.20c	9.75 ± 0.69a	8.92 ± 0.42a	7.12 ± 0.44b	10.62 ± 0.20c
	TP	128.13 ± 1.59c	80.73 ± 0.50a	103.5 ± 1.78b	184.58 ± 1.99d	82.92 ± 2.72a	102.74 ± 1.51b
OH• (μmol TE/g DW)	FP	53.69 ± 0.84c	56.26 ± 1.29c	32.92 ± 1.90a	82.64 ± 1.70d	36.13 ± 3.16a	42.02 ± 3.44b
	BP	14.17 ± 0.55a	14.54 ± 0.43a	13.26 ± 0.50a	13.23 ± 0.74a	13.90 ± 0.29a	13.43 ± 0.71a
	TP	67.86 ± 1.39b	70.80 ± 1.72b	46.18 ± 2.40a	95.87 ± 2.44c	50.03 ± 3.45a	56.45 ± 4.15a
FRAP (mM FeS(II) E/g DW)	FP	43.77 ± 1.48c	31.70 ± 0.33a	38.82 ± 0.22b	84.72 ± 3.29d	29.58 ± 1.31a	37.06 ± 1.79b
	BP	2.90 ± 0.18a	4.35 ± 0.31c	3.41 ± 0.16b	2.95 ± 0.12a	2.23 ± 0.14a	3.84 ± 0.03b
	TP	46.67 ± 1.66b	36.05 ± 0.64a	43.23 ± 0.38b	87.67 ± 3.41c	31.81 ± 1.45a	40.90 ± 1.82b

Different lowercase letters (a–e) mean statistically significant differences following different samples (*p* < 0.05) at the same status. FP, free phenolic; BP, bound phenolic; TP, total phenolic. N.D. not detected.

**Table 4:** The analysis results of the main phenolic analytes’ ligands docking into *α*-glucosidase.

Main Phenolics	C-Score	T-Score	PMF-Score	CHEM-Score	G-Score	D-Score
Quercetin	5	6.37	−137.893	−27.110	−173.998	−143.148
Rutin	4	5.94	−260.712	−31.241	−310.716	−278.108
Kaempferol-3-*O*-rutinoside	4	4.68	−147.036	−26.576	−210.020	−220.257
Dihydromyricetin	4	5.50	−167.849	−29.871	−287.447	−247.370

**Table 5:** Correlation matrix between the major phenolic compounds and the investigated bio-activities.

Analytes	Correlations Matrix				
	DPPH	ABTS^+^	OH•	FRAP	GIA (IC_50_)
FP	0.990***	0.983**	0.851*	0.998***	−0.671*
BP	0.583*	0.932**	0.803*	0.947**	−0.604*
FF	0.994***	0.981**	0.765*	0.974**	−0.723*
BF	0.731*	0.572*	0.686*	0.601*	−0.622*
GA	0.933**	0.931**	0.924**	0.970**	−0.585
4-HA	0.455	0.513	0.546*	0.535*	−0.478
5-CA	0.545	0.587*	0.750*	0.577*	−0.324
SA	0.208	0.371	0.198	0.151	−0.401
DIM	0.934**	0.938**	0.877*	0.912**	−0.765*
RUT	0.959**	0.963**	0.633*	0.921**	−0.803*
FA	0.550*	0.251	0.299	0.092	−0.396
KAE-3-*O*-RUT	0.992**	0.985**	0.804*	0.982**	−0.551*
QUE	0.952*	0.699*	0.895**	0.895*	−0.715*
API	0.478	0.375	0.245	0.382	−0.348
KAE	0.453	0.402	0.800*	0.558	−0.618*

* Correlation was significant at the 0.05 level (two-tailed). ** Correlation was significant at the 0.01 level (two-tailed). *** Correlation was significant at the 0.001 level (two-tailed).

## Supplementary Materials

The following are available online at https://www.mdpi.com/2076-3921/8/12/606/s1, Table S1: Identification of free and bound phenolic compounds from buckwheat samples by HPLC-DAD-ESI-qTOF/MS. FP, free phenolic; BP, bound phenolic.

## Abbreviations

BW	buckwheat
DW	dried weight
TPC	total phenolic content
TFC	total flavonoid content
FP	free phenolic
BP	bound phenolic
FF	free flavonoid
BF	bound flavonoid
GAE	gallic acid equivalents
RE	rutin equivalents
TE	trolox equivalents
DPPH	1,1-diphenyl-2-picrylhydrazyl
ABTS	2, 2-azino-bis (3-ethylbenzothiazoline-6-sulfonic acid) diammonium salt
FRAP	ferric reducing/antioxidant power
TPTZ	2,4,6-tris(2-pyridyl)-s-triazine
