# Supplementary material for: Free and Bound Phenolics of Buckwheat Varieties: HPLC Characterization, Antioxidant Activity, and Inhibitory Potency towards α-Glucosidase with Molecular Docking Analysis
Source: Antioxidants (Basel). 2019 Nov 29;8(12):606. doi: 10.3390/antiox8120606 (PMC6943536; doi:10.3390/antiox8120606)
Supplement: Supplementary file 1 [file antioxidants-08-00606-s001.pdf]

antioxidants

Table 1. Identification of free and bound phenolic compounds from buckwheat samples by HPLC-DAD-ESI-qTOF/MS. FP, free phenolic; BP, bound phenolic.

Peak No.	RT	Molecular ion (*m*/*z*)	MS/MS fragments	MW	Chemical formula	Compound	FP	BP
1	4.67	171.02 $[M+H]^+$	171.02, 127.10	170	$C_7H_6O_5$	Gallic acid	√	√
2	11.25	137.03 $[M-H]^-$	137.03, 98.02	138	$C_7H_5O_3$	4−Hydroxybenzoic acid	√	√
3	12.72	353.09 $[M-H]^-$	353.09, 191.01	354	$C_{16}H_{18}O_9$	5-Caffeoylquinic acid	√	√
4	13.12	197.07 $[M-H]^-$	197.07, 193.01	198	$C_9H_{10}O_5$	Syringic acid	√	√
5	15.37	321.10$[M+H]^+$	321.10, 299.20	320	$C_{15}H_{12}O_8$	Dihydromyricetin	-	√
6	17.21	611.26 $[M+H]^+$	611.29, 466.37, 303.21, 160.29, 137.13	610	$C_{27}H_{31}O_{16}$	Rutin	√	√
7	19.31	193.06 $[M-H]^-$	193.06, 134.01	194	$C_{10}H_{10}O_4$	Ferulic acid	√	√
8	20.82	595.17 $[M+H]^+$	595.17, 287.13	594	$C_{27}H_{30}O_{15}$	Kaempferol-3-O-rutinoside	√	√
9	34.53	303.05 $[M+H]^-$	303.05, 135.29	302	$C_{15}H_{10}O_7$	Quercetin	√	√
10	38.87	270.24 $[M+H]^+$	271.24, 137.12	270	$C_{15}H_{10}O_7$	Apigenin	√	√
11	39.89	287.05 $[M+H]^+$	287.05, 153.01	286	$C_{15}H_{10}O_6$	Kaempferol	√	√
